# Biological Potential and Mechanism of Prodigiosin from *Serratia marcescens* Subsp. *lawsoniana* in Human Choriocarcinoma and Prostate Cancer Cell Lines

**DOI:** 10.3390/ijms19113465

**Published:** 2018-11-04

**Authors:** Dan Li, Jun Liu, Xin Wang, Di Kong, Wei Du, Hongbo Li, Chung-Yun Hse, Todd Shupe, Dongpo Zhou, Kai Zhao

**Affiliations:** 1 Engineering Research Center of Agricultural Microbiology Technology, Ministry of Education, Heilongjiang University, Harbin 150080, China; docor1005@163.com (D.L.); tianronghaise@126.com (X.W.); zhoudp0451@163.com (D.Z.); 2 Key Laboratory of Microbiology, College of Heilongjiang Province, School of Life Science, Heilongjiang University, Harbin 150080, China; kongdi1110@126.com (D.K.); duweihappy123@163.com (W.D.); hljbobo@tust.edu.cn (H.L.); 3 School of Pharmaceutical Sciences, Collaborative Innovation Center for Diagnosis and Treatment of Infectious Diseases, Tsinghua University, Beijing 100084, China; 4 College of Food and Biological Engineering, Qiqihar University, Qiqihar 161006, China; 5 USDA Forest Service Southern Research Station, Adhesive and Composite Laboratory, Pineville, LA 71360, USA; chse@fs.fed.us; 6 Louisiana Forest Products Development Center, School of Renewable Natural Resources, Louisiana State University Agricultural Center, Baton Rouge, LA 70803, USA; tfshupe@gmail.com

**Keywords:** *Serratia marcescens* subsp. *lawsoniana*, prodigiosin, secondary metabolites, human choriocarcinoma cell lines and human prostate cancer cell lines, anticancer activities and mechanism

## Abstract

Tripyrrole molecules have received renewed attention due to reports of numerous biological activities, including antifungal, antibacterial, antiprotozoal, antimalarial, immunosuppressive, and anticancer activities. In a screen of bacterial strains with known toxicities to termites, a red pigment-producing strain, HDZK-BYSB107, was isolated from *Chamaecyparis lawsoniana*, which grows in Oregon, USA. Strain HDZK-BYSB107 was identified as *Serratia marcescens* subsp. *lawsoniana*. The red pigment was identified as prodigiosin using ultraviolet absorption, LC-MS, and 1H-NMR spectroscopy. The bacterial prodigiosin had an inhibitory effect on both Gram-negative and Gram-positive bacteria. The main objective of this study was to explore the anticancer activities and mechanism of strain HDZK-BYSB107 prodigiosin by using human choriocarcinoma (JEG3) and prostate cancer cell lines (PC3) in vitro and JEG3 and PC3 tumor-bearing nude mice in vivo. In vitro anticancer activities showed that the bacterial prodigiosin induced apoptosis in JEG3 cells. In vivo anticancer activities indicated that the prodigiosin significantly inhibited the growth of JEG3 and PC3 cells, and the inhibitory activity was dose and time dependent. The anticancer efficacy of the bacterial prodigiosin on JEG3 and PC3 cells, JEG3 and PC3 tumor exhibited a correlation with the down regulation of the inhibitor of IAP family, including XIAP, cIAP-1 and cIAP-2, and the activation of caspase-9 and caspase-3 accompanied by proteolytic degradation of poly (ADP-ribose)-polymerase. The expressions of P53 and Bax/Bcl-2 in JEG3 and PC3 cells were significantly higher than in untreated groups. Our results indicated that the bacterial prodigiosin extracted from *C. lawsoniana* is a promising molecule due to its potential for therapeutic applications.

## 1. Introduction

The many commercially available anticancer drugs can be classified by origin as either chemical synthetic drugs or natural drugs derived from various kinds of organisms [1,2,3]. Natural medicine for cancer therapy has proved to be effective and less toxic on normal cells, with fewer side effects [4]. Prodigiosin did not cause death in vitro to lymphocytes at effective concentrations (<100 nM) and also did not show toxicity in vivo to lymphoid organs at effective dosages (10 and 30 mg/kg) [5]. The discovery of anticancer drugs has mainly resulted from screening of natural products and their analogs [4,6]. Some natural plant metabolites are believed to have anticancer properties; these include several pigments, quinines, and alkaloids [7,8,9,10]. Secondary metabolites from microorganisms are more practical for development as therapeutic agents [11,12,13].

As an anticancer drug, prodigiosin shows its anticancer activity by inhibiting or activating some signaling pathways that have not been clearly understood before [14,15]. Prodiginines (PGs) are a family of tripyrrole red pigments receiving increasing interest because of their numerous biological activities, including antifungal, antibacterial, antiprotozoal, antimalarial, immunosuppressive, and anticancer activities [16,17,18]. Specially, prodigiosin has been effective in tumor cell inhibition and cell apoptosis induction [19,20,21,22]. Prodigiosin is a secondary metabolite produced from *Serratia species* and other unrelated microbial strains, such as *Streptomyces griseoviridis*, *Pseudomonas magnesiorubera*, *Vibrio species*, and other marine bacteria [23,24,25]. Some of the enzymes involved in the biosynthetic pathways that produce prodigiosin are now known, and some of the corresponding genes have been identified and cloned [26,27], but the biosynthetic pathway is still poorly understood. Prodigiosin production in some producer organisms, such as *Serratia* and *Streptomyces*, is now well understood, as well as its physiology and regulation. However, its biological role in these organisms remains unclear.

Prodigiosin is known to induce apoptosis in a broad range of cancer cell lines. Importantly, its pro-apoptotic effect is selective against malignant cells irrespective of p53 status and multidrug resistance, rendering prodigiosin a promising anticancer agent [28,29]. Understanding the action mechanism of bacterial prodigiosin is essential for drug development and is required for the identification and characterization of the still unidentified cell target. The prodigiosin can inhibit Wnt/β-catenin signaling and exerts anticancer activity in breast cancer cell [20] and can reactivate p53 family-dependent transcriptional activity in p53-deficient human colon cancer cells [21]. In particular, the action mechanism of bacterial prodigiosin and triggers apoptosis in human choriocarcinoma and prostate cancer cell lines has not been reported. The anticancer activities displayed by prodigiosin are thought to be the result of several modes of action: caspase-dependent and -independent induction of apoptosis, activation of protein kinase pathways, and induction of cell-cycle arrest. *Serratia marcescens-*derived prodigiosin had been reported that it can increase caspase-3 levels in prodigiosin-treated acute lymphoblastic leukemia cells [30]. The family of caspases is a major mediator of apoptosis in cancer cells, and apoptosis is initiated by the activation of a set of death effector cysteine proteases called caspases [31,32]. X-linked inhibitor of apoptosis protein (XIAP), cellular inhibitors of apoptosis 1 and 2 (cIAP 1 and cIAP 2) belong to the inhibitor of apoptosis proteins (IAPs), which represent a family of endogenous caspase inhibitors and also play an important role in inducing apoptosis of cancer cells [33,34,35]. It has been reported that the undecylprodigiosin (UP) could cause a marked decrease of the levels of antiapoptotic XIAP while enhancing the levels of proapoptotic BIK, consequently favoring induction of apoptosis in human breast carcinoma cell lines MCF-7 BT-20, MDA-MB-231 and T47D. In addition, Ho et al. found that cells with functional p53 (MCF-7, T47D) or mutant p53 (BT-20, MDA-MB-231) were both susceptible to UP’s cytotoxicity [29]. The inhibitor of apoptosis (IAP) family proteins is involved in mechanisms of resistance to apoptosis in various cancer cells [36,37,38]. In addition, the poly(ADP-ribose) polymerases (PARPs) are a family of enzymes that catalyze the transfer of ADP-ribose from nicotinamide adenine dinucleotide onto acceptor proteins. Among this family, which consists of at least 17 members, PARP-1 is the most widely investigated. PARP1 has a role in repair of single-stranded DNA breaks, and PARP1 knockout shows no negative phenotype and no increased incidence of tumor formation [39,40,41]. It has been reported that prodigiosin can activate endoplasmic reticulum stress cell death pathway in human breast carcinoma cell lines. In addition, prodigiosin down-regulated Bcl-2 in a CHOP-dependent manner. Importantly, restoration of Bcl-2 expression blocked prodigiosin-induced PARP cleavage and greatly enhanced the survival of prodigiosin-treated cells, suggesting that CHOP-dependent Bcl-2 suppression mediates prodigiosin-elicited cell death [42]. As we achieve a deeper understanding of the molecular mechanisms underlying induced JEG3 and PC3 cells death, the role of mitochondria-cytochrome c pathway in cancer treatment in vitro and in vivo will be elucidated.

*Chamaecyparis lawsoniana* is known to have significantly high levels of natural durability and termiticidal activity due to the inherently high attractive content of its heartwood [43,44]. In this paper, we described the isolation of a prodigiosin-producing bacterium, HDZK-BYSB107, collected from Port Orford Cedar (POC), *C*. *lawsoniana*, in Oregon, USA. The prodigiosin-producing strain was identified as *Serratia marcescens* subsp. *lawsoniana*. We extracted and purified a red pigment from this strain and identified it as prodigiosin by ultraviolet absorption analysis, mass spectrographic analysis, LC-MS, and NMR spectroscopy. Therefore, to explore the anticancer activities and mechanism of the bacterial prodigiosin, we performed this study using human choriocarcinoma (JEG3) and prostate cancer cell lines (PC3) in vitro, and JEG3 and PC3 tumor-bearing nude mice in vivo. Our results suggested that the bacterial prodigiosin had strong antibacterial, anticancer, and proapoptotic activities against cancer cells and raises the possibility of its use as a chemotherapeutic drug in future.

## 2. Results

### 2.1. Identification of the Prodigiosin-Producing Strain HDZK-BYSB107

#### 2.1.1. Morphological Characterization

**Figure 1:**
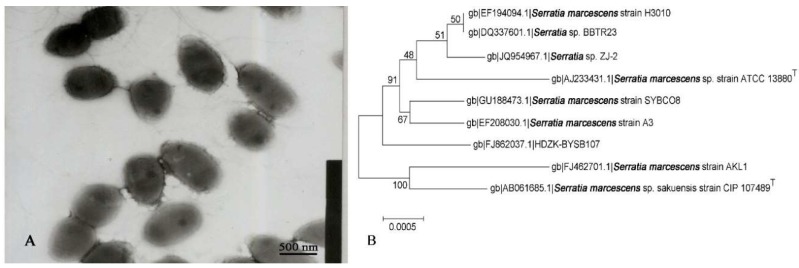
Identification of strain HDZK-BYSB107. (**A**) Scanning electron micrograph of strain HDZK-BYSB107; (**B**) Phylogeny tree generated for strain HDZK-BYSB107 based on the 16S DNA sequence.

Colonies of strain HDZK-BYSB107 were dark red in color with a glossy, oval, nontransparent, and dry appearance on the surface, without obvious fold-like protuberances. They had regular edges and slightly prominent centers on beef extract and peptone medium. Strain HDZK-BYSB107, standard strain, and two reference strains were Gram-negative and had capsules (Figure 1A and Table S1).

As shown in Table S1, the morphological characteristics of strain HDZK-BYSB107 were closest to those of *Serratia marcescens*, but with some differences. Cells of strain HDZK-BYSB107 were substantially smaller in size than those of the standard strain ATCC8100 and the reference strains HBU-01 and FU-01 (0.7–0.8 × 2.0–2.5 µm), with cell sizes of 0.45–0.55 × 0.75–0.85 μm (Figure 1A and Table S1). In addition, the lowest and optimum growth temperatures of strain HDZK-BYSB107 were 10 and 28 °C, respectively, on beef extract and peptone medium. In contrast, the optimum growth temperature of both the standard strain and reference strains was 37 °C, and the lowest growth temperature for both was 15 °C. Strain HDZK-BYSB107, standard strain and reference strains were all Gram-negative, and cells were mostly found in pairs, with occasional single cells.

#### 2.1.2. Physiological and Biochemical Characteristics of Strain HDZK-BYSB107

As shown in Table S2, the physiological and biochemical characteristics of strain HDZK-BYSB107 were similar to those of the standard strain and reference strains, with some differences. For example, strain HDZK-BYSB107 was able to produce arginine hydrolase, grow in 6% NaCl and 20% sucrose, and use xylose and rhamnose to produce acid as sole carbon sources, whereas the standard and reference strains could not do any of these. In addition, strain HDZK-BYSB107 was not able to use urea as a nitrogen source.

#### 2.1.3. Molecular Analysis

The 16S rDNA of strain HDZK-BYSB107 was successfully amplified by PCR, with an expected size of 1468 bp. After sequencing, the newly identified sequences were deposited into GenBank (Accession number FJ862037). In homology searches against the GenBank and proprietary bacterial DNA databases, the sequences of strain HDZK-BYSB107 were found to share 99% similarity with those of *S. marcescens*. A phylogenetic relationship was established through alignment and classical analysis of nucleotide sequences among bacterial species (Figure 1B), which revealed strain HDZK-BYSB107 to be most closely related to the genus *Serratia*. According to the phylogenetic analysis, strain HDZK-BYSB107 was classified as *genus Serratia* and as a subspecies of *S. marcescens*.

### 2.2. Isolation, Purification, and Identification of Prodigiosin from Strain HDZK-BYSB107

**Figure 2:**
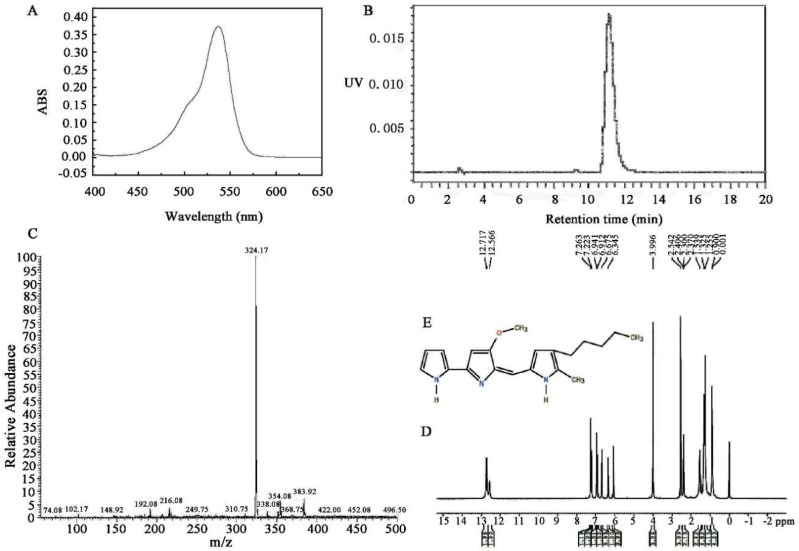
Biochemical characterization of prodigiosin from strain HDZK-BYSB107. (**A**) UV-visible absorption spectrum of the purified pigmented extract; (**B**) HPLC analysis of the purified pigmented extract; (**C**) ELI-MS analysis of the purified pigmented extract; (**D**) 1H-NMR spectrum of prodigiosin; E: The estimated structural formula of prodigiosin.

Using column chromatography, a red liquid fraction was acquired from a culture supernatant of strain HDZK-BYSB107. UV-visible absorption spectroscopy on an extract of strain HDZK-BYSB107 obtained using acidic methanol revealed strong absorption in the range of 420–560 nm, with maximum absorption at 533 nm (Figure 2A). Upon purification using chromatography, only the band with the strongest absorption at 533 nm was observed. The methanol extracts of non-pigmented strains did not absorb in the range of 400–600 nm.

The purity (99%) and quantity of pigment extracted from strain HDZK-BYSB107 were determined by high performance liquid chromatography coupled with mass spectrometry (HPLC-MS). During the HPLC analysis of the purified sample, the major product eluted at 11.125 min, similar to what has been reported elsewhere for prodigiosin (Figure 2B) [45]. As shown in Figure 2C, a molecular ion peak M + H of prodigiosin at *m*/*z* 324, which corresponds to the molecular weight of prodigiosin (C20H25N3O, 323 kD), was detected. The results for molecular mass were similar to those previously reported in the literature [45].

The structure of strain HDZK-BYSB107 prodigiosin was further confirmed by high-field 1H-NMR spectroscopy (Figure 2D). The 1H-NMR spectrum of baterial prodigiosin shows the peaks corresponding to chemical shifts at δ 7.22 ppm, δ 6.94 ppm, δ 3.99 ppm, δ 2.38 ppm, δ 1.25 ppm, and δ 0.88 ppm assigned to the carbon atoms based on the structure of batrerial prodigiosin presented in Figure 2E.

### 2.3. Physical and Chemical Properties of Prodigiosin from Strain HDZK-BYSB107

The prodigiosin from strain HDZK-BYSB107 was readily soluble in alcohols and DMSO and relatively stable in an acid environment (pH = 3). In addition, it exhibited anti-oxidant and anti-reducing activity and was thermally stable.

### 2.4. Activity Assays on the Prodigiosin from Strain HDZK-BYSB107

#### 2.4.1. Antimicrobial Activity Assay

**Figure 3:**
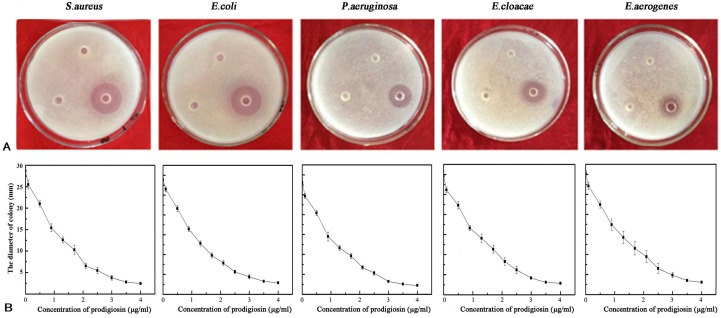
Antimicrobial activity assay of prodigiosin from strain HDZK-BYSB107 by (**A**) Disc-diffusion and (**B**) Dot inoculation methods.

The bacterial prodigiosin extracted from strain HDZK-BYSB107 had the antimicrobial activities against *S. aureus*, *E. coli*, *P. aeruginosa*, *E. cloacae*, and *E. aerogenes*, with inhibition zone diameters of 24.02 ± 0.18, 22.74 ± 0.21, 18.32 ± 0.30, 14.52 ± 0.05 and 10.10 ± 0.17 mm, respectively, when 0.1 mg of extract was applied. As shown in Figure 3A, the strongest inhibition was seen against *S. aureus*, and the weakest was against *E. aerogenes*.

The relationship between the concentration of pigmented metabolite and microbial cytotoxic effect was determined using dot inoculation assays. As shown in Figure 3B, the IC_50_ (concentration of bacterial prodigiosin required to destroy 50% of cells) was 1.82 μg/mL for *S. aureus*, 1.81 μg/mL for *E. coli*, 1.76 μg/mL for *P. aeruginosa*, 1.72 μg/mL for *E. cloacae*, and 1.65 μg/mL for *E. aerogenes*. The results were similar using a disc-diffusion method; as the concentration of bacterial prodigiosin increased, cytotoxicity increased.

#### 2.4.2. In Vitro Apoptotic Activity Assay

**Figure 4:**
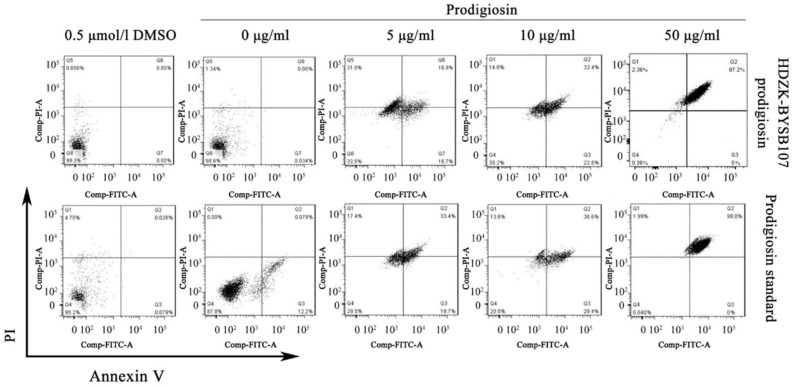
The apoptotic activity of prodigiosin from strain HDZK-BYSB107 in vitro. Flow cyclometer for the dual staining of Annex V and PI. Annex V+/PI− cells indicates the cells undergoing early apoptosis. Q1: mechanically damaged cells; Q2: late apoptotic cells; Q3: early apoptotic cells; Q1: living cells.

To determine the anticancer effect of the bacterial prodigiosin on JEG3 cells, JEG3 cells undergoing apoptosis after the treatment of various concentration of bacterial prodigiosin or prodigiosin standard were prepared by the Annexin V-propidium iodide (PI) apoptosis detection kit according to the manufacturer’s instructions. As shown in Figure 4, with the increasing prodigiosin concentration, the apoptosis demonstrated a dose-dependent relationship in the early apoptotic cells. It could be seen that the HDZK-BYSB107 prodigiosin induced apoptosis of JEG3 cells with a similar apoptotic ratio in early apoptosis phase compared to the prodigosin standard (18.7% vs. 19.7%) after the treatment of 5 μg/mL prodigiosin. Apoptosis was induced with a similar apoptotic ratio in early apoptosis phase compared to the prodigosin standard (22.6% vs. 29.4%) in the 10 μg/mL prodigiosin treatment group. We also found that there was only 0.452% survival of JEG3 cells in the 50 μg/mL bacterial prodigiosin group, which was nearly similar to 0.040% survival JEG3 cells in the prodigosin standard treatment group. The superior apoptosis effect for the prodigiosin group was consistent with the prodigosin standard group. These results showed that we obtained the bacterial prodigiosin with high purity, which has a similar ability of inducing apoptosis in cancer cells to prodigiosin standard. Furthermore, with the increasing prodigiosin concentration, the apoptotic rate was dramatically increased. Since then, the trend of apoptosis was generally stable (Figure S1).

#### 2.4.3. In Vivo Anticancer Activity Assay

**Figure 5:**
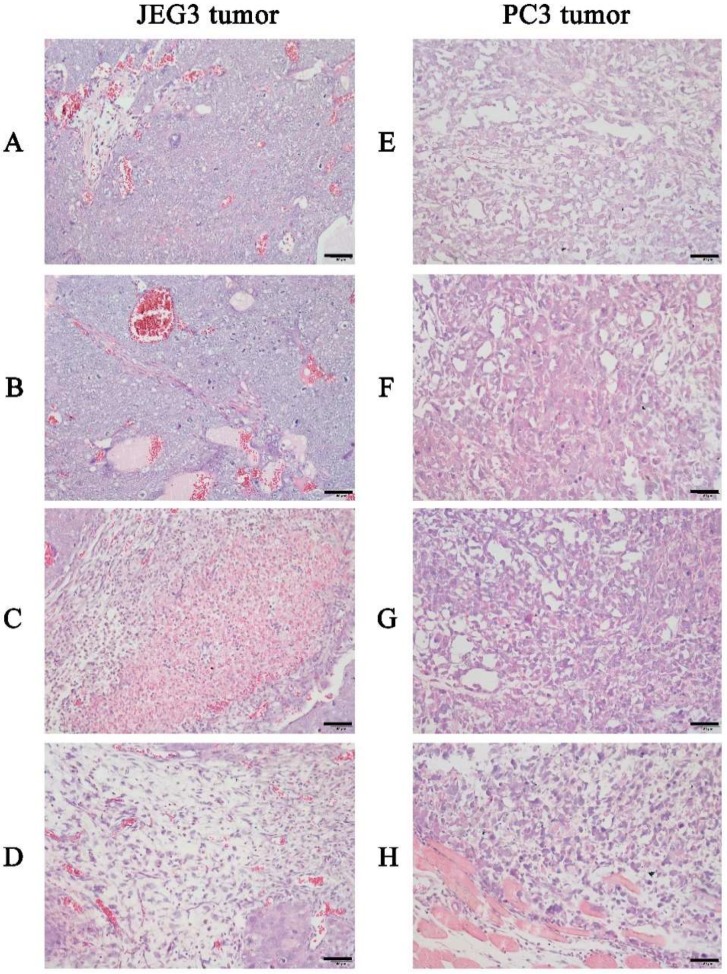
In vivo anticancer activity of prodigiosin from strain HDZK-BYSB107. Tumor tissues were stained with H & E and analyzed under a light microscope after injection with different concentrations of prodigiosin for 16 d. **A** and **E** is untreated mice; **B** and **F**, **C** and **G**, **D** and **H** are mice injected with 50, 100, and 250 μg/kg prodigiosin, respectively. Scale bar = 50 μm.

**Figure 6:**
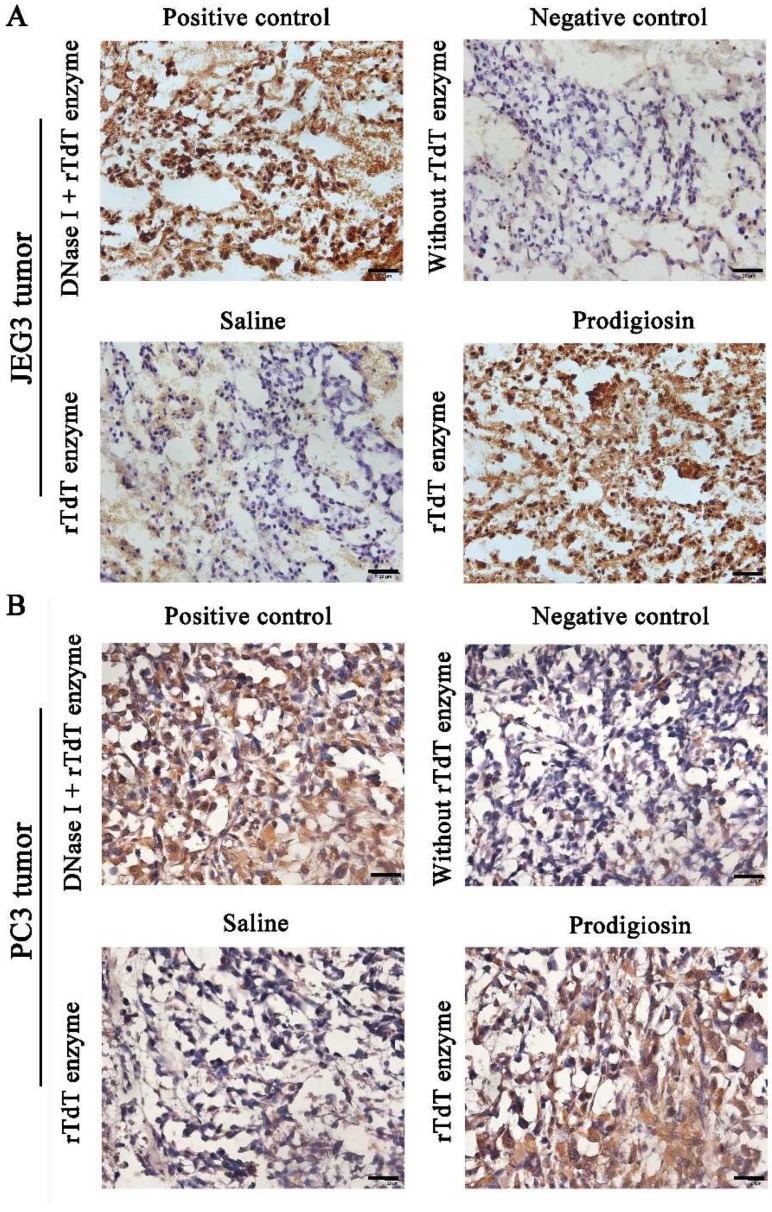
Apoptosis induced by the HDZK-BYSB107 prodigiosin via Tunnel assay. Apoptosis of JEG3 cells (**A**) and PC3 cells (**B**) induced by the HDZK-BYSB107 prodigiosin in vivo. The positive control was treated by DNase I and rTdT enzyme and the negative control was treated without rTdT enzyme. Saline was used as a control compared to the prodigiosin treatment group. Scale bar = 20 μm.

To assess the anticancer activities of the bacterial prodigiosin in vivo, we obtained female BALB/c tumor-bearing nude mice (8 weeks old) caused by hypodermic injection of JEG3 cancer cells or PC3 cancer cells and administered the bacterial prodigiosin to the nude mice via the tail vein. Tumor volume began to decrease shortly after injection, and the treatments produced significant dose-dependent decreases in tumor tissue volume and weight (*p* < 0.05) (Table 1 and Table 2). To further investigate the effect on tumors, the orthotropic tumors and tissues with suspected metastatic tumors were resected and sectioned, stained with hematoxylin and eosin, and analyzed under a light microscope (Figure 5). As can be seen from Figure 6, the cancer cells exhibited atypia and a dense and disordered arrangement with visible interstitial cells. The bacterial prodigiosin significantly inhibited the growth of JEG3 (Figure 5A–D) and PC3 (Figure 5E–H) tumors, and with the increasing of the bacterial prodigiosin concentration, the difference became more significant (*p* < 0.01); the inhibitory activities were dose and time dependent (Table 1 and Table 2).

The further induction of apoptosis or other forms of cell death in JEG3 tumor and PC3 tumor obtained from the mice injected with the HDZK-BYSB107 prodigiosin for 16 days was evaluated by TUNEL assay. As shown in Figure 6, compared with the positive control treated with DNase I, the negative control without rTdT Enzyme incubation and the saline groups, the JEG3 cells (Figure 6A) and PC3 cells (Figure 6B) in the HDZK-BYSB107 prodigiosin-treated group showed significantly increased apoptosis (*p* < 0.01, Figure 6).

### 2.5. Anticancer Mechanism of the Prodigiosin from Strain HDZK-BYSB107

**Figure 7:**
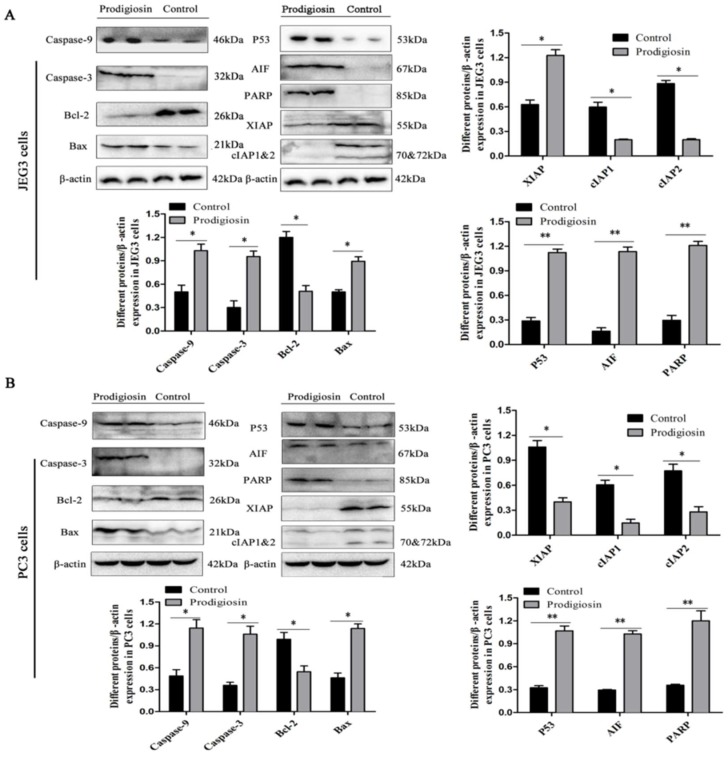
Western blot assay of the bacterial prodigiosin-induced apoptosis. Extracts of JEG3 cells (**A**) and PC3 cells (**B**) were used for anticancer mechanism analysis. Expression levels of Caspase-9, Caspase-3, Bcl-2, Bax, P53, AIF, PARP and IAP family proteins in JEG3 and PC3 cells treated with the 50 µg/mL of aerial prodigiosin for 24 h. “*” and “**” indicate statistically significant difference at *p* < 0.05 and *p* < 0.01 (Student’s *t* test), respectively.

**Figure 8:**
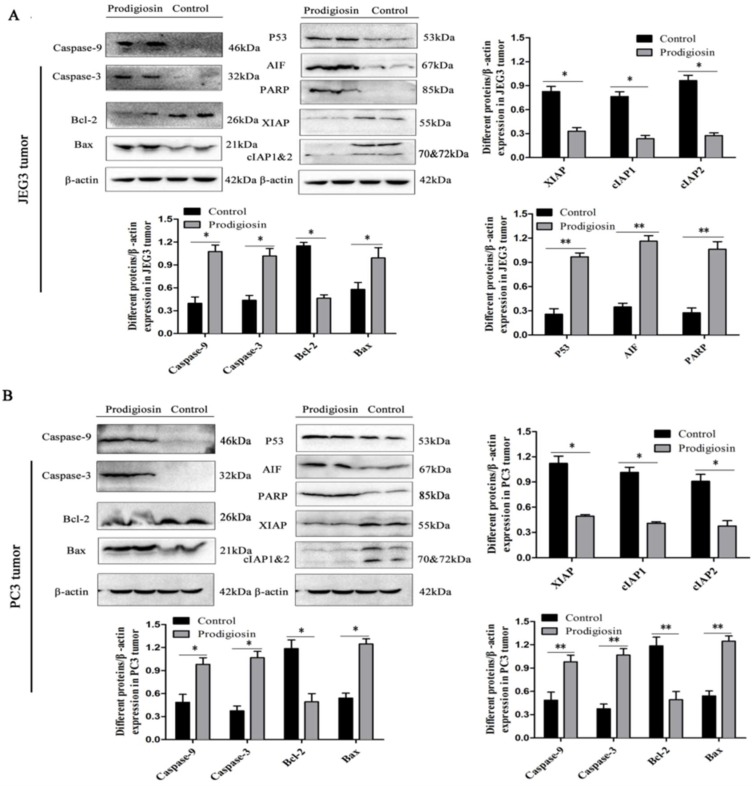
Western blot assay of the bacterial prodigiosin-induced apoptosis. Extracts of JEG3 tumor (**A**) and PC3 tumor (**B**) were used for anticancer mechanism analysis. Expression levels of Caspase-9, Caspase-3, Bcl-2, Bax, P53, AIF, PARP and IAP family proteins in JEG3 and PC3 tumor treated with the 50 μg/mL of bacterial prodigiosin for 24 h. “*” and “**” indicate statistically significant difference at *p* < 0.05 and *p* < 0.01 (Student’s *t* test), respectively.

To verify the anticancer mechanism of HDZK-BYSB107 prodigiosin, the JEG3 and PC3 cells and tumors were treated with the bacterial prodigiosin (50 μg/mL) and analyzed by western blotting. The activation of PARP and release of mitochondrial factors, including AIF (apoptosis inducing factor), were found both in the cells in vitro and tumor in vivo. As investigated in JEG3 (Figure 7A) and PC3 cells (Figure 7B), and in JEG3 tumor (Figure 8A) and PC3 tumor (Figure 8B), the activated PARP and AIF were not clearly found in control groups; however, the stronger bands (67 kD and 85 kD) were obviously observed in the bacterial prodigiosin-treated groups. The expressions of PARP and AIF in the JEG3 cells or PC3 cells, and JEG3 tumor or PC3 tumor treated with the bacterial prodigiosin were significantly higher than those in the control cells or tumors (*p* < 0.01, Figure 7 and Figure 8).

**Figure 9:**
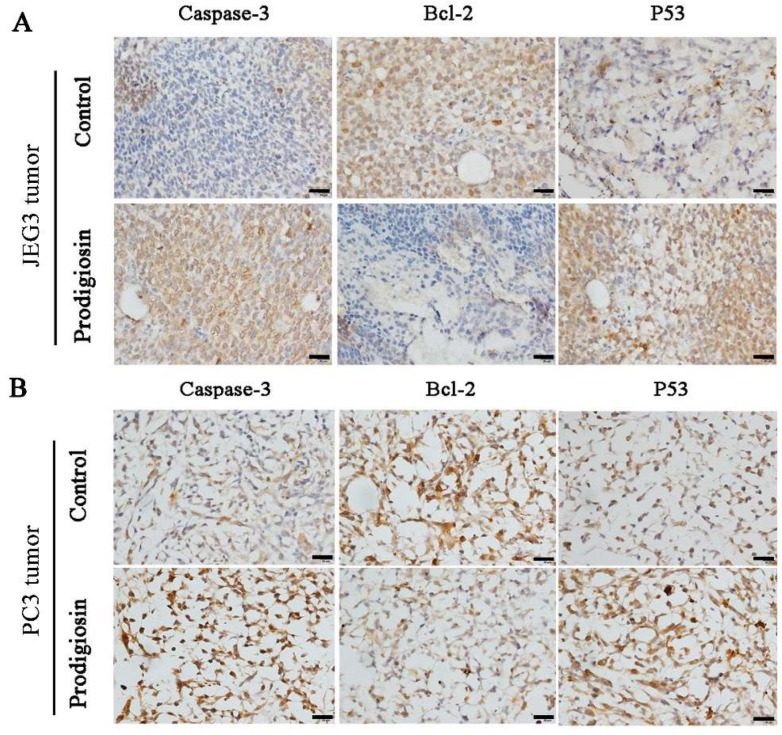
Immunocytochemistry assay of the bacterial prodigiosin-induced apoptosis. JEG3 (**A**) and PC3 (**B**) tumor slice collected from 50 μg/mL of bacterial administrated mice were used for immunocytochemistry analysis of Caspase-3, Bcl-2 and P53 in vivo. Scale bar = 20 μm.

To investigate whether the bacterial prodigiosin promotes cancer cells apoptosis via the mitochondria-cytochrome c pathway, including caspase, Bax and Bcl-2, western blotting was performed after cells and tumors were treated with the 50 μg/mL of bacterial prodigiosin for 48 h. In the control cells or tumors groups, activated caspase-3 and caspase-9 were not found, while the 32 and 46 kD bands indicated active caspase-3 and caspase-9 were found in the 50 μg/mL of bacterial prodigiosin group (Figure 7, Figure 8 and Figure 9). However, the expression of Bcl-2 significantly decreased in the group treated by the bacterial prodigiosin (*p* < 0.05, Figure 7, Figure 8 and Figure 9). During the bacterial prodigiosin induction, Bax protein expression increased while Bcl-2/Bax decreased. In other words, the bacterial prodigiosin may increase the ratio of Bax to Bcl-2 in JEG3 cells, PC3 cells, JEG3 tumor and PC3 tumor. Additionally, the IAPs were detected by western blotting, and the results showed that the bacterial prodigiosin significantly decreased the expressions of XIAP and cIAP1&cIAP2 protein in the JEG3 and PC3 cancer cells as compared to the control group cells (*p* < 0.05, Figure 7 and Figure 8). Simultaneously, after the treatment with the bacterial prodigiosin, the expression level of p53 was significantly up-regulated compared to the untreated cells (*p* < 0.01, Figure 7, Figure 8 and Figure 9).

## 3. Discussion

Previous studies have reported that prodigiosin or its analogue-producing strains include *Serratia*, *Streptomyces*, *Hahella*, and *Vibrio* [23,24,25]. Strain HDZK-BYSB107 belongs to *Serratia*, and it is noteworthy that the strain HDZK-BYSB107 is an endophytic bacterium from *C. lawsoniana*. This is the first report of a prodigiosin-producing strain from *C. lawsoniana* with much more prodigiosin output of 0.656 g/L compared to *Zooshikella rubidus* S1-1 (0.048 g/L) and *Hahella chejuensis* KCTC 2396 (0.028 g/L) [46,47]. In addition, the antimicrobial activities of bacterial prodigiosin against *S. aureus* and *E. coli* with 24.02 ± 0.18, 22.74 ± 0.21 mm were stronger than the prodigiosin from *Serratia marcescens* SU-10 with 20 and 16 mm [48].

The study of prodigiosin and its biosynthesis has been developed for more than fifty years since it was originally identified as an anticancer drug with pronounced features [48,49,50]. Most of the commercially available anticancer drugs are chemically synthesized and have substantial side effects [1]. The cytotoxic activity of prodigiosin against a wide range of human cancer cell lines and relatively lower toxicity toward nonmalignant cells is unique [48,49,50]. Although this study did not address the mechanisms of biological activities of prodigiosin, many previous studies have suggested the mechanisms for the anticancer effect of prodigiosin [15,16,17]. Studies have shown that a bacterial bioactive metabolite (undecylprodigiosin, UP) produced by *Streptomyces* and *Serratia*, exerted a potent cytotoxicity against all breast carcinoma cell lines in a dose- and time-dependent manner. Notably, UP-induced apoptosis was blocked by the pan-caspase inhibitor z-VAD-fmk, further indicating the involvement of caspase activity [29]. The activation of caspases-9, -8, and -3 in prodigiosin-treated Jurkat cells in a dose-response study has been carefully examined [51], in addition to the activation of caspases-9, and caspase-3 in the bacterial prodigiosin-treated JEG3 cells in a dose-responsive manner, which was similar to above undecylprodigiosin treated Jurkat cells. However, in the present study, JEG3 cells and PC3 cells were firstly used to investigate the mechanism of the bacterial prodigiosin on tumor cells in vitro and in vivo, respectively. These results also contribute to potential applications of prodigiosin, especially anticancer potency, and suggest that prodigiosins are potential therapeutic drugs.

Prodigiosin has much less toxicity than other anticancer drugs, but further research is needed before use as a therapeutic drug for clinical use [49]. Although the molecular mechanisms of cytotoxic effects of prodigiosin on malignant cells remain largely unknown, it has been shown that prodigiosin induces apoptosis in many human cancer cell lines [28]. Four possible mechanisms have been suggested, including prodigiosin as DNA cleavage agents, as mitogen-activated protein kinase regulators, and as pH modulators and as cell cycle inhibitors [52]. Studies have found that apoptosis mainly has two independent apoptotic pathways [53], one is mitochondria-cytochrome c path, another is activation path through death receptors. In the present study, the apoptotic response induced by the bacterial prodigiosin was mainly through the mitochondria-cytochrome c pathway. Cytochrome c, which is released into the cytoplasm, activates procaspase-9 that has the ability to activate effective caspases including caspase-3 and also leads to apoptosis. In most of the apoptotic processes, caspase-8 is involved in the extrinsic pathway, caspase-9 is involved in the intrinsic pathway, and caspase-3 plays a pivotal role in the terminal and execution phase of apoptosis [54]. The bacterial prodigiosin induced caspase-3 and caspase-9 activation, and the subsequent activation of PARP.

The Bcl-2 family has a significant anti-apoptotic function through interaction with a large number of proteins. The Bcl-2 family can be divided in two types in accordance with its structure and function, which includes anti-apoptotic and pro-apoptotic pathways. The anti-apoptotic pathway includes Bcl-xL and Bcl-2, while the other pathway is pro-apoptotic, such as Bax. Bcl-2 and Bax are the two most essential members in the Bcl-2 family, which are closely associated with tumors [55,56]. In order to compare with the original mechanisms, we confirmed that the expression of Bcl-2 in JEG3 and PC3 tumors declined by prodigiosin, while the expression of Bax increased. To date, six members have been found in the IAP family in humans, including XIAP, cIAP1, cIAP2, survivin, NIAP, and BRUCE. Inhibitors of apoptosis (IAPs) proteins were initially characterized by their ability to directly bind and inhibit apoptotic caspases. The study of the mechanism of IAP-induced apoptosis inhibition showed that IAPs played a very essential role in apoptosis inhibition by inhibiting the activity of the caspase family, such as caspase-3, caspase-7 and caspase-9, but had no effect on the activity of caspase-1, caspase-6 and caspase-8 [57]. The most important biological effect of p53 protein is to increase the expression of the p21 gene. In the cell cycle, the p53 protein repairs damaged DNA or chromosome by preventing cells in G1 phase from entering into the S phase. When DNA or chromosomes suffer severe damage, p53 can trigger the apoptosis mechanism to remove damaged cells so as to regulate and promote the function of radiotherapy and chemotherapy agents [58]. In agreement with other reports, our results also showed that the percentage of apoptotic cells did not change with increasing incubation time or prodigiosin concentration [17]. Contrasted with the results of in vivo anticancer analysis, a conclusion can be drawn that the rapid cell death induced by the bacterial prodigiosin cannot be explained only by caspase-dependent pathways; other mechanisms also contribute to the observed cell death.

## 4. Materials and Methods

### 4.1. Strains and Cells

Strain HDZK-BYSB107 (stored in the China Center for Type Culture Collection, CCTCC NO 209163) is a wild-type strain that can produce prodigiosin. The strain HDZK-BYSB107 was isolated from POC, *C. lawsoniana.* POC was collected in Oregon, USA.

A standard strain of *Serratia marcescens*, ATCC8100, and two reference strains, *S. marcescens* HBU-01 and FU-01, were used for the identification of strain HDZK-BYSB107. The standard strain ATCC8100 was obtained from the China General Microbiological Culture Collection Center (Beijing, China). The reference strain HBU-01 was obtained from Humboldt University, Germany, and the reference strain FU-01 was obtained from University of Florida, USA.

*Staphylococcus aureus*, *Escherichia coli*, *Pseudomonas aeruginosa*, *Enterobacter cloacae* and *Enterobacter aerogenes* were used for the antimicrobial assay of prodigiosin from strain HDZK-BYSB107. The strains were stored in the Key Laboratory of Microbiology, School of Life Science in Heilongjiang University, China.

JEG3 human choriocarcinoma cell lines and PC3 human prostate cancer cell lines were provided by the Laboratory for Reproductive Health, Shenzhen Institutes of Advanced Technology of the Chinese Academy of Science. JEG3 and PC3 cell lines were cultured as monolayers in Dulbecco’s modified Eagle’s medium supplemented with 10% (*v*/*v*) calf serum, 100 units of penicillin/mL, 100 μg of streptomycin/mL, and 10 mmol/L HEPES (pH 7.2) at 37 °C and 5% CO_2_.

### 4.2. Isolation and Culture of Strain HDZK-BYSB107

The phloem of POC bark was selected and treated with 75% ethanol for 3–5 min, and the surfaces were then washed with sterile distilled water. The disinfected wood was cut into 1.5 × 1.0 × 0.5 cm sections and placed into beef extract and peptone media plates containing nystatin and cultured at 37 °C for 2–3 d. Uncut wood was treated using the same procedures and rolled on the surface of the beef extract and peptone media plates as blank controls.

Colonies that can produce red pigment in the media were transferred to beef extract and peptone media containing 3 g/L beef extract, 10 g/L peptone, and 5 g/L NaCl at 37 °C for 10–24 h. A purified single colony was obtained by the standard plate-streaking technique. The strain was named HDZK-BYSB107 and was stored in the Key Laboratory of Microbiology, School of Life Sciences, Heilongjiang University. All media components were purchased from either Becton Dickinson (Sparks, MD, USA) or Sigma Aldrich (Munich, Germany).

### 4.3. Identification of Strain HDZK-BYSB107

#### 4.3.1. Morphological Examination of Strain HDZK-BYSB107

Strain HDZK-BYSB107 was activated at 28 °C on a plate of beef extract and peptone media. The mycelia were inoculated at three different locations on a 9 cm plate and incubated at 28 °C for 18–24 h. Strain HDZK-BYSB107 was detected and identified at the genus and species levels based on morphological characteristics [45]. The morphology of strain HDZK-BYSB107 was examined by both light microscopy and transmission electron microscopy, and somatic diameters were measured after Gram staining, crystal violet staining, negative staining, spore staining, and silver staining of flagella. Digital micrographs of colonies were taken with a Coolpix 995 camera (Nikon, Tokyo, Japan).

#### 4.3.2. Physiological and Biochemical Characteristics of Strain HDZK-BYSB107

Physiological and biochemical characteristics were examined as described previously [59,60]. Each test was performed on three replicates.

#### 4.3.3. Molecular Analysis of Strain HDZK-BYSB107

Culture and collection of mycelia were carried out as described previously [61]. For sequence analysis, DNA was extracted from strain HDZK-BYSB107 and identified in accordance with previously described methods [62].

The 16S rDNA sequences were amplified by polymerase chain reaction (PCR) with the primer pair 5′-CCGGATCCAGAGTTTGATCCTGGTCAGAACGAACGCT-3′ and 5′-CGGGATCCTACGGCTAC CTTGTTACGACTTCACCCC-3′. PCR reactions were carried out with one cycle of heat treatment at 94 °C for 10 min, a total of 35 cycles of denaturation at 94 °C for 1 min, annealing at 55 °C for 30 s, extension at 72° C for 1.5 min, and a final extension at 72 °C for 7 min. The PCR products were stored at 4 °C, later analyzed by 0.8% agarose gel electrophoresis, and then sequenced. Sequencing of the PCR products was performed by Sangon Co. Ltd. (Shanghai, China). 16S rDNA was sequenced in both directions with an ABI PRISM 377-18 DNA Sequencer (Applied Biosystems, Foster City, CA, USA) according to the manufacturer’s instructions. The sequences were submitted to GenBank for homology searches using BLAST (http//ncbi.nim.nih.gov). The sequences of 16S rDNA were aligned with those of related bacterial strains retrieved from GenBank using CLUSTAL X. A phylogenetic tree was constructed from the evolutionary distances by PHYLIP, version 3.57c (distributed by J. Felsenstein, Department of Genetics, University of Washington, Seattle, WA, USA).

### 4.4. Preparation and Isolation of Fermentation broth from Strain HDZK-BYSB107

After activation on a slant tube of beef extract and peptone media at 28 °C for 18–24 h, strain HDZK-BYSB107 was inoculated into 200 mL of beef extract and peptone media and cultivated for 24 h at 28 °C with rotation at 160 r/min. Then, 10 mL of the seed culture medium was transferred to 500 mL of fermentation medium containing 5 g/L maltose, 10 g/L peptone, 5g/L NaCl, and 0.3 g/L NaOAC and cultured for 72 h at 28 °C with rotation at 160 r/min. Bacteria were then harvested by centrifugation at 8000 r/min for 10 min at 4° C. The strain HDZK-BYSB107 prodigiosin was extracted by shaking the pellet with acidic methanol (methano1: l mol / l HCl = 24: l) and centrifuging at 3000 r/min for 5 min at room temperature. The supernatant was dried under vacuum, and the pigment was redissolved in DMSO, divided into aliquots, and stored at −20 °C.

### 4.5. Isolation, Purification, and Identification of Prodigiosin from Strain HDZK-BYSB107

The absorption spectrum of the isolated prodigiosin was determined using an Ultrospec 3300 pro UV/Visible Spectrophotometer (Amersham Biosciences) at a wavelength range of 400–650 nm. Prodigiosin was purified [63]. Briefly, after evaporation under vacuum of the acidic methanol solvent (4% 1 mol/L HCl in 96 mL methanol), atmospheric pressure liquid chromatography of the extract was performed on silica gel with chloroform and methanol as solvents. The eluted fractions were pooled, and the chloroform/methanol extract was vacuum dried, redissolved in H_2_O, and lyophilized. The isolated pigment was redissolved in methanol and analyzed by electrospray ionization mass spectrometry (ESI-MS) using a Thermo Fisher LCQ fleet mass spectrometer (Thermo Fisher Scientific, Waltham, MA, USA). The isolated pigment was re-purified by subsequent semi-preparative high-performance liquid chromatography (HPLC) on a Shimadzu instrument (Kyoto, Japan). A Nucleosil C18 reverse phase column (4.6 × 250 mm, 5 µm) was used with a 0% to 100% linear gradient over 30 min (solvent A = 10 mmol/L ammonium acetate, solvent B = acetonitrile). The eluate was monitored using a diode-array UV detector (SPD-MlOAVP, Shimadzu, Kyoto, Japan) and by ESI-MS.

After repeated injections, the pooled fractions containing the major peaks were vacuum evaporated, redissolved in H_2_O, lyophilized, and characterized by ESI-MS and 1H-NMR analyses. High-resolution ESI-MS spectra were recorded in the *m*/*z* range 100–3200 in positive ion mode, with a 4000 V ion source potential and a 140 V fragmentor potential [41]. 1H NMR spectra were recorded on a Bruker Avance II spectrometer (Bruker, Germany) operating at 400 and 100.6 MHz. DEPT, and 2D experiments (1H–1H COSY, HSQC, HMBC, and NOESY) were run on the same instrument with the usual pulse sequences. All NMR spectra were measured at 25 °C in CDCl_3_ with TMS as an internal standard. The amount of the internal standard used was adjusted so that it corresponded to the highest integral of the compound quantified.

### 4.6. Activity Assays on Prodigiosin from Strain HDZK-BYSB107

#### 4.6.1. Antimicrobial Activity Assay

The antimicrobial activities of purified strain HDZK-BYSB107 prodigiosin on *E. aerogenes*, *S. aureus*, *P. aeruginosa*, *E. coli*, and *E. cloacae* were studied using a previously reported disc-diffusion method [24]. Briefly, late stationary phase cells of test microorganisms were spread on agar plates, and sterile paper discs (6 mm diameter) were applied to the surface. Different concentrations of the bacterial prodigiosin extracted by methanol in the same volume of solvent were applied to the discs. The loaded discs were placed on the surface of the media and left for 30 min at room temperature for compound diffusion. A mixture of DMSO and methanol was used as the vehicle solvent and as a negative control. The plates were incubated for 18–24 h at 28 °C. Zones of inhibition were recorded in millimeters, and the experiments were conducted in triplicate.

The antimicrobial activities of the bacterial prodigiosin were also determined by dot inoculation assays. Prodigiosin samples were assayed in triplicate at concentrations of 0.1, 0.5, 0.9, 1.3, 1.7, 2.1, 2.5, 3.0, 3.5, and 4.0 μg/mL in the media. The test organisms were spot seeded on the culture media in triplicate, and colony sizes were determined every 24 h.

#### 4.6.2. In Vitro Anticancer Activity Assay

To test the apoptotic JEG3 cells induced by the bacterial prodigiosin, apoptosis assay was performed by the Annexin V/FITC/PI apoptosis assay kit (Molecular Probes, Thermo Fisher Scientific, Waltham, CA, USA) and analysed by using FACS flow Cytometer (BD Biosciences, San Diego, CA, USA). The phosphatidyl serine (PS) on the cell surface was used to identify the cell apoptosis. Annexin V has high affinity to PS and can be taken up by apoptotic cells or necrotic cells. Therefore, propidium iodide (PI) and Annexin were used to examine necrotic cells and apoptotic cells (early and late apoptosis). Briefly, the JEG3 cells were diluted to 2 × 10^6^/mL and seeded in 6-well plate (2 mL/well) with three duplicates. The cells were treated with different concentrations (0, 5, 10, 50, 100 μg/mL) of bacterial prodigiosin or prodigiosin standard, respectively, for overnight incubation at 37 °C, and were incubated with 0.5 μmol/L DMSO as a control. The cells in 6-well plate were incubated for another 24 h following the bacterial prodigiosin and prodigiosin standard treatment. The cells were subsequently washed with ice-cold phosphate buffer saline (PBS) three times, trypsinized, centrifuged and re-dispersed in a binding buffer. The cells in six groups were treated with 5 μL of propidium iodide (PI) and 5 μL of Annexin and incubated for 15 min at room temperature. Then, the mixed volume was analyzed by the FACS flow Cytometer (BD Biosciences, San Diego, CA, USA) and Diva software.

#### 4.6.3. In Vivo Anticancer Activity Assay

Female BALB/C nude mice (8 weeks old) were maintained at 23 ± 2 °C with a relative humidity of 50–60% under a 12: 12 h light–dark cycle and provided food and water ad libitum. Prior to performing the experimental procedures, mice were matched for body weight. Then, 4 × 10^5^/mL JEG3 and PC3 cells were injected subcutaneously into the mice and the experiments were carried out until the tumor volume grew up to 200 mm^3^, and the size of subcutaneous tumors was measured after 16 d. All animals were randomly divided into groups (*n* = 4): the control group, and the treatment groups, which received the bacterial prodigiosin doses of 50, 100 or 250 μg/kg. The bacterial prodigiosin was administered by intratumoral injection daily for 16 days, thereby representing a repeated-dose study. At 16 days after treatment, the mice were sacrificed and tumor tissue was removed, collected, and weighed. Tumor growth rates were calculated by comparing the sizes of tumors before and after the bacterial prodigiosin injection. The tumor tissues were fixed in 4% PBS-formaldehyde and processed. Histological analysis of tumor tissues was performed according to the protocol described before [64]. Tissue sections were prepared on a Microm CM3050S (Leica, Wetzlar and Mannheim, Germany) with the microtome chamber chilled to −22 °C and the specimen holder set at −18 °C. The sections were prepared at a thickness of 5 μm and then stained with hematoxylin-eosin (H&E) for microscopic observation (Leica IX71, Leica, Wetzlar and Mannheim, Germany) as described before [64]. Body weight was evaluated every two days and at the end of treatment. Care of laboratory animals and animal experimentation were performed in accordance with animal ethics guidelines with the protocols approved by Animal Ethics Committee of the Shenzhen Institutes of Advanced Technology, Chinese Academy of Sciences (SIAT), China (SIAT-IRB-170406-YYS-FXJ-A0350) for Animal Care and Use, and the date of approval was 6 April 2017.

### 4.7. TUNEL Assay

Apoptotic cells were detected using the DeadEnd™ Colorimetric TUNEL System (Promega Inc., USA) according to the manufacturer’ s instructions. Briefly, deparaffinized JEG3 and PC3 tumor sections were incubated with terminal deoxynucleotidyl transferase (TdT) enzyme in equilibration buffer and biotinylated nucleotide mix in a humidified chamber at 37 °C for 60 min, and tagged nucleotides were detected using the peroxidase substrate, hydrogen peroxide, and the stable chromogen, diaminobenzidine (DAB). Diaminobenzene was used as the chromogen and hematoxylin as the nuclear counterstain. Sections were then dehydrated, cleared and mounted examined under a phase-contrast microscope (Olympus CKX 41; Olympus, Hachioji, Japan). To quantify apoptotic cells, TUNEL-positive cells were counted using ImageJ (NIH) by examining ten random fields for each tumor tissue per experimental condition.

### 4.8. Anticancer Mechanism of the Prodigiosin from Strain HDZK-BYSB107

The paraffin sections of JEG3 tumor and PC3 tumor were prepared for immunocytochemistry assay. The slides were incubated with caspase-3, p53 and Bcl-2 primary antibody (Abcam Inc., USA, 1:100) at 4 °C overnight and IgG/HRP secondary antibody (Beijing Biosynthesis Inc., Beijing, China, 1:200) for 30 min at room temperature. Then, the staining was visualized by using a DAB chromogen solution. Samples of JEG3 cells, PC3 cells, JEG3 tumor and PC3 tumor for western blotting were homogenized in ice-cold RIPA buffer (Santa Cruz Biotechnology, Inc., Santa Cruz, CA, USA) containing protease inhibitors. The protein concentration of the samples was determined using a BCA protein assay kit (Thermo Fisher Scientific, Waltham, MA, USA). Proteins in each sample were separated by sodium dodecyl sulfate-polyacrylamide gel electrophoresis (SDS-PAGE) and were transferred onto polyvinylidene difluoride (PVDF) membranes (Millipore, Billerica, MA, USA). Nonspecific binding was blocked with 5% non-fat skim milk in Tris buffered saline with 0.05% Tween-20 (TBST) at room temperature for 1 h. Membranes were incubated overnight with rabbit polyclonal anti-IGFBP5 antibody (1:1000; Abcam, Cambridge, UK), rabbit polyclonal anti-caspase-3 antibody, rabbit polyclonal anti-caspase-9 antibody (1:1000; Santa Cruz Biotechnology, Inc.), rabbit polyclonal anti-P53 antibody (1:500; Abcam, Cambridge, UK), rabbit polyclonal anti-Bcl-2 antibody, rabbit polyclonal anti-Bax antibody, rabbit polyclonal anti-AIF antibody, rabbit polyclonal anti-cIAP1&cIAP2 antibody, rabbit polyclonal anti-XIAP antibody (1:1000; Abcam, Cambridge, UK) and rabbit polyclonal anti-PARP antibody (1:1000; Santa Cruz Biotechnology, Inc.) at 4 °C for 12 h. After washing with TBST, membranes were incubated with horseradish peroxidase-conjugated goat anti-rabbit IgG (1:2000; Amersham Biosciences, Tokyo, Japan) at room temperature for 1 h. The bound secondary antibodies were visualized with Thermo Scientific Pierce ECL Western Blotting Substrate (Thermo Scientific Pierce, Rockford, IL, USA) according to the manufacturer’s instructions. Signal intensities of bands were analyzed by Image J software (National Institutes of Health, Bethesda, MD, USA), and the relative protein levels were calculated by comparison with β-actin (1:5000; Abcam, Cambridge, UK), used as a loading control. All western blot data shown were representative of three independent experiments.

### 4.9. Statistical Analysis

All data are presented as the mean value ± S.D. Comparison of difference between two groups was evaluated by Student’s *t*-test. Kruskal-Wallis one-way analysis of variance (ANOVA) was employed to evaluate the statistical differences among the different groups with SPSS 19.0 software (IBM SPSS, Chicago, IL, USA). The difference between groups with *p*-value of <0.05 or <0.01 was considered statistically significant.

## 5. Conclusions

Natural anticancer drugs have been proven effective and less toxic for cancer therapy and are being used in combination with other anticancer drugs against a variety of cancers. We extracted and purified a red pigment from strain HDZK-BYSB107 and identified it as prodigiosin. In addition, the antimicrobial activities, anticancer activities and mechanism of the bacterial prodigiosin were evaluated. Different antimicrobial activities against *S. aureus*, *E. coli*, *P. aeruginosa*, *E. cloacae*, and *E. aerogenes* were observed. However, the anticancer activity in vitro and in vivo tumor growth inhibition were observed with the treatments of HDZK-BYSB107 prodigiosin. Those above results for isolated HDZK-BYSB107 prodigiosin were shown to be able to effectively inhibit tumor growth. Together with its excellent anticancer activity, the anticancer mechanism was further verified by JEG3 and PC3 cells. We confirmed that the bacterial prodigiosin promotes cancer cell apoptosis via up regulation of caspase-3, caspase-9, Bax, P53, AIF, PARP protein expression and down regulation of Bcl-2, XIAP and cIAP1&cIAP2 protein expression in JEG3 and PC3. Thus, we concluded that the HDZK-BYSB107 prodigiosin could be considered as a non-toxic anticancer drug in the near future.

## Figures and Tables

**Table 1:** The effects of different concentrations of prodigiosin from strain HDZK-BYSB107 on growth of JEG3 and PC3 tumors in BALB/C nude mice.

Groups	Dosage of Prodigiosin (μg/kg)	Tumor Volume before Treatment (cm^3^)	Tumor Volume after 2 d Treatment (cm^3^)	Tumor Volume after 9 d Treatment (cm^3^)	Tumor Volume after 16 d Treatment (cm^3^)
JEG3 cells	0	0.207 ± 0.031	0.225 ± 0.014	0.253 ± 0.017	0.281 ± 0.022
PC3 cells		0.212 ± 0.132	0.237 ± 0.168	0.261 ± 0.052	0.295 ± 0.085
JEG3 cells	50	0.206 ± 0.142	0.225 ± 0.012	0.252 ± 0.005	0.279 ± 0.013
PC3 cells		0.212 ± 0.174	0.236 ± 0.053	0.259 ± 0.031	0.294 ± 0.047
JEG3 cells	100	0.202 ± 0.016 ^a^	0.205 ± 0.043 ^a^	0.192 ± 0.192 ^a^	0.180 ± 0.142 ^a^
PC3 cells		0.216 ± 0.194 ^a^	0.208 ± 0.018 ^a^	0.195 ± 0.087 ^a^	0.182 ± 0.104 ^a^
JEG3 cells	250	0.211 ± 0.067 ^a^	0.169 ± 0.069 ^b^	0.152 ± 0.172 ^b^	0.144 ± 0.067 ^b^
PC3 cells		0.209 ± 0.037 ^a^	0.163 ± 0.041 ^b^	0.155 ± 0.054 ^b^	0.149 ± 0.127 ^b^

Compared with the control group, values within the same column with the different lower-case letters (a, b) in the superscript are significantly different (*p* < 0.05).

**Table 2:** JEG3 and PC3 tumor inhibition by the different concentrations of prodigiosin from strain HDZK-BYSB107.

Groups	Dosage of Prodigiosin (μg/kg)	Tumor Weight (g)	Tumor Inhibition Rate (%)
JEG3 cells	0	0.85 ± 0.13	
PC3 cells		0.87 ± 0.21	
JEG3 cells	50	0.84 ± 0.34	1.17 ± 0.19
PC3 cells		0.86 ± 0.02	1.14 ± 0.25
JEG3 cells	100	0.77 ± 0.01^*^	9.41 ± 0.14^*^
PC3 cells		0.81 ± 0.03^*^	6.80 ± 0.32^*^
JEG3 cells	250	0.64 ± 0.05^**^	24.71 ± 0.29^**^
PC3 cells		0.69 ± 0.13^**^	20.69 ± 0.17^**^

* Statistically significant difference at *p* < 0.05; ** Statistically significant difference at *p* < 0.01.

## Supplementary Materials

Supplementary materials can be found at http://www.mdpi.com/1422-0067/19/11/3465/s1.
