# Supplementary material for: Biological Potential and Mechanism of Prodigiosin from Serratia marcescens Subsp. lawsoniana in Human Choriocarcinoma and Prostate Cancer Cell Lines
Source: Int J Mol Sci. 2018 Nov 4;19(11):3465. doi: 10.3390/ijms19113465 (PMC6274741; doi:10.3390/ijms19113465)
Supplement: Supplementary file 1 [file ijms-19-03465-s001.pdf]

Biological Potential and Mechanism of Prodigiosin from *Serratia marcescens* Subsp. *lawsoniana* in Human Choriocarcinoma and Prostate Cancer Cell Lines

Dan Li [1,2,3,†], Jun Liu [4,*,†], Xin Wang [1,2,†], Di Kong [2], Wei Du [2], Hongbo Li [2], Chung-Yun Hse [5], Todd Shupe [6], Dongpo Zhou [1,2] and Kai Zhao [1,2,*]

1. Engineering Research Center of Agricultural Microbiology Technology, Ministry of Education, Heilongjiang University, Harbin 150080, China; docor1005@163.com (D.L.); zhoudp0451@163.com (D.Z.)
2. Key Laboratory of Microbiology, College of Heilongjiang Province, School of Life Science, Heilongjiang University, Harbin 150080, China; tianronghaise@126.com (X.W.); kongdi1110@126.com (D.K.); duweihappy123@163.com (W.D.); hljbobo@tust.edu.cn (H.L.)
3. School of Pharmaceutical Sciences, Collaborative Innovation Center for Diagnosis and Treatment of Infectious Diseases, Tsinghua University, Beijing 100084, China
4. College of Food and Biological Engineering, Qiqihar University, Qiqihar 161006, China
5. USDA Forest Service Southern Research Station, Adhesive and Composite Laboratory, Pineville, LA 71360, USA; chse@fs.fed.us
6. Louisiana Forest Products Development Center, School of Renewable Natural Resources, Louisiana State University Agricultural Center, Baton Rouge, LA 70803, USA; tfshupe@gmail.com

* Correspondence: liujun3117@163.com (J.L.); zybin395@126.com (K.Z.); Tel.: +86-452-273-8743 (J.L.); +86-451-8660-8586 (K.Z.)

† These authors contributed equally to this work.

Table S1. Shape and growth temperature of strain HDZK-BYSB107.

Characteristics	Strain HDZK-BYSB107	Standard strain ATCC 8100	Reference strains	
			Strain HBU-01	Strain FU-01
Shape of strain body	oval	short rod	short rod	short rod
Arrangement of strain body	pairs or single	single	single	single
Cell size (am)	0.45-0.55×0.75-0.85		0.7-0.8×2.0-2.5	0.7-0.8×2.0-2.5
Colony size (mm)	2.2			
Colony color	dark red	red	light red	light red
Lowest growth temperature	10° C	15° C	15° C	15° C
Optimum growth temperature	28° C	37° C	37° C	37° C
Gram staining	—	—	—	—
Capstone	+	+	+	+

"+" positive reaction; "—" indicates negative reaction.

Table S2. Physiological and biochemical properties of strain HDZK-BYSB107.

Characteristics	Strain HDZK-BYSB107	Standard strain ATCC8100	Reference strains	
			Strain HBU-01	Strain FU-01
Anaerobic growth	＋	＋	＋	＋
Motility	＋	＋	＋	＋
Peroration & cattail	＋	＋	＋	＋
Growth with 2% Clonal	＋	＋	＋	＋
Growth with 6% Clonal	＋	—	—	—
V-P reaction	＋	＋	＋	＋
In dole	＋		＋	＋
Methyl red	—	—	—	—
Cit rate utilization	＋	＋	＋	＋
H_2S	—	—	—	—
Gelatin hydrolysis (22° C)	＋	＋	＋	＋
Elysian declaratory	—	—	—	—
Arline hydro lase	＋	—	—	—
Earthiness declaratory		＋	＋	＋
Marinate utilization	—	—	—	—
Starch hydrolysis	＋	＋	＋	＋
Urea utilization	—	＋	＋	＋
Growth with 2% sucrose	＋	＋	＋	＋
Growth with 10% sucrose	＋	＋	＋	＋
Growth with 20% sucrose	＋	—	—	—
Pigment	red	pink	pink	pink
Broth with 40% cholera	—	—	—	—
Acid production from glucose	＋	＋	＋	＋
Acid production from sucrose	＋	＋	＋	＋
Acid production from fructose	＋	＋	＋	＋
Acid production from salience	＋	＋	＋	＋
Acid production from loosely	＋	—	—	—
Acid production from inseparable	—	—	—	—
Acid production from rhinoceros	＋	—	—	—
Acid production from Sorbonne	—	—	—	—
Acid production from absorbing	—	—	—	—
Acid production from raffishness	—	—	—	—
Acid production from Mann	—	—	—	—
Acid production from mannish	—	—	—	—

"＋" positive reaction; "—" negative reaction.

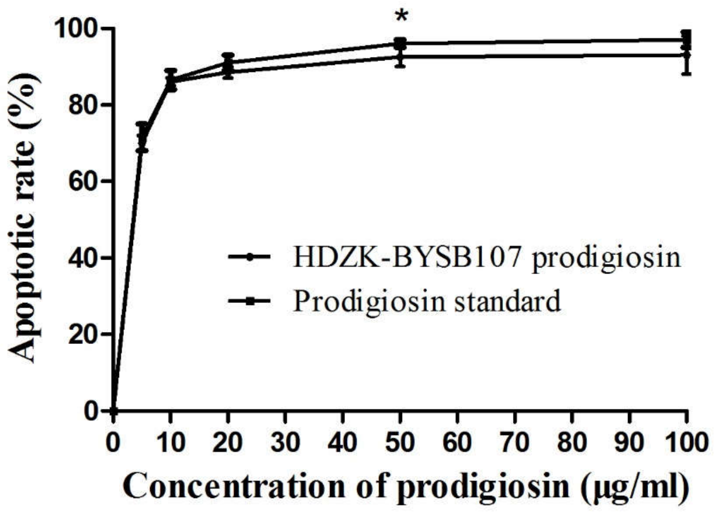

Figure S1. Apoptotic rate of JEG3 cells treated with the different concentration of strain HDZK-BYSB107 prodigiosin or prodigiosin standard.
